# Teacher support as predictors of Chinese EFL learners’ classroom flow: the mediating role of academic self-efficacy

**DOI:** 10.3389/fpsyg.2024.1452146

**Published:** 2024-09-27

**Authors:** Wenting Gong, Chuang Xu

**Affiliations:** ^1^School of Educational Science, Hunan Normal University, Changsha, China; ^2^School of International Studies, Hunan Institute of Technology, Hengyang, China; ^3^School of Marxism, Hunan Institute of Technology, Hengyang, China

**Keywords:** teacher support, classroom flow, academic self-efficacy, EFL learning, structural equation modeling

## Abstract

Informed by social support theory and control-value theory, this study investigates the predictive role of teacher support on classroom flow among English as a Foreign Language (EFL) learners and the mediating effect of academic self-efficacy. A survey was conducted among 557 Chinese university EFL learners to gather relevant data. Descriptive statistics indicate that the participants exhibited a moderate level of classroom flow and exhibited significant variations based on gender and grade. Analysis using structural equation modeling revealed that teacher support has a noteworthy, positive predictive impact on EFL learners’ classroom flow. Furthermore, academic self-efficacy serves as a partial mediator between teacher support and classroom flow, with instrumental and emotional teacher support showing the strongest indirect effects. Notably, various dimensions of teacher support, aligning with students’ psychological needs as outlined by self-determination theory, have unique predictive effects on both classroom flow and academic self-efficacy. Appraisal and informational teacher support have the greatest predictive influence on classroom flow among the teacher support considered. The findings enhance understanding of the dynamics between teacher support, academic self-efficacy, and classroom flow, offering practical implications for creating autonomy-supportive educational environments that can elevate EFL learners’ engagement and academic achievements.

## 1 Introduction

The emergence of positive psychology in the field of second language acquisition (SLA) has shed new light on the importance of flow experience in educational contexts. Flow, or flow experience, as defined by Csikszentmihalyi (1988), refers to a psychological state characterized by high concentration and engagement that leads to advanced performance on certain tasks. This phenomenon is increasingly recognized as relevant in the Chinese university second language (L2) learning environment, where the quest for academic excellence is deeply ingrained in the educational ethos. In the specific context of English as a Foreign Language (EFL) classrooms in China, the pursuit of flow is particularly pertinent. As Czimmermann and Piniel (2016) suggest, the flow experience can significantly enhance student engagement and foster a deeper sense of accomplishment, which are pivotal to language acquisition. However, varying levels of flow can elicit distinct academic emotions. Students who encounter negative flow tend to experience comparable levels of boredom and anxiety, whereas those in a state of positive flow report similar degrees of enjoyment, as observed by Wang and Huang (2022). This has prompted a surge in research aimed at identifying the antecedents of flow within language educational settings. Empirical evidence has underscored the pivotal role of individual factors, such as learning motivation and self-efficacy (Piniel and Albert, 2017) alongside environmental variables like task characteristics (Egbert, 2003), in cultivating flow experiences. The influence of teacher support, a critical environmental factor, has been recognized as particularly influential in shaping academic practices and student performance (Tao et al., 2022; Wang, 2022), which greatly contribute to fostering students’ flow experience. Egbert (2003) posited that the learning tasks assigned by teachers can elicit varying degrees of flow. He defined tasks as “high flow” if two-thirds or more of the students reported experiencing it, and “low flow” if flow was perceived by one-third or fewer students. Additionally, teachers, through their supportive roles, can significantly impact students’ self-efficacy by adjusting the classroom atmosphere, changing their instructional strategies, and improving teacher-student interaction (Chong et al., 2018) as well.

Despite the acknowledged significance of teacher support, the specific effects of various forms of teacher support on EFL learners’ flow experiences remain under-explored, especially within the Chinese university context. Furthermore, the potential mediating role of academic self-efficacy in this relationship has not been thoroughly investigated. Academic self-efficacy, defined as an individual’s belief in their capabilities to achieve educational goals (Elias and MacDonald, 2007), is a well-documented predictor of positive learning outcomes and experiences (Honicke and Broadbent, 2016). However, its role in mediating the relationship between teacher support and flow experiences among EFL learners in Chinese universities is less understood. Hence, this study aims to address these research gaps by investigating the predictive influence of teacher support on the classroom flow experiences of Chinese university EFL learners, who are the largest EFL populations worldwide (Wang et al., 2021). It also intends to clarify the mediating role of academic self-efficacy in the relationship between teacher support and flow experiences. In addition, numerous studies suggest that certain demographic variables, including gender, age, and academic major, may exert varying influences on students’ classroom flow (e.g., Hsieh et al., 2016; Dewaele and MacIntyre, 2019; Wang, 2022). Thus the following research questions (RQs) are proposed:

1. What is the prevalent level of flow experience within Chinese university EFL classrooms? Do Chinese university EFL learners of different genders, academic years, and majors differ in terms of flow experience?
2. In what ways does teacher support contribute to enhancing the flow experience of Chinese EFL students?
3. Does academic self-efficacy mediate the relationship between teacher support and flow experience in the context of Chinese university EFL classrooms?

## 2 Literature review

### 2.1 Theoretical background: an integrated perspective

Social support theory (Cohen and Wills, 1985) can be utilized to anticipate the association between teacher support and students’ flow experience. It examines how assistance and support provided by members of one’s social network (such as family, friends, colleagues, or teachers) influence an individual’s mental health, coping mechanisms, and overall wellbeing. There are two primary models that interweave and interact to explain how social support influences individuals’ psychology and behavior. The direct-effect model of social support indicates that social support has a universal gain effect, and increasing social support can improve individual mental health and provide positive emotional experiences. The buffering model, on the other hand, suggests that that social support can have indirect effects by influencing individuals’ cognition and coping styles, such as providing positive solutions to problems and healthy behavior. In the EFL learning context, teachers can help students cope with pressures and difficulties by providing a safe and supportive environment, enabling them to maintain their flow experience even in the face of challenges.

Apart from social support theory, control-value theory offers a complementary theoretical perspective for examining the role of academic self-efficacy in the relationship between teacher support and flow experience. According to control-value theory, achievement emotions are triggered by the interaction of control and value perceptions regarding academic tasks (Pekrun, 2006). When learners perceive that their efforts are likely to result in successful task completion - a perception closely tied to their academic self-efficacy - they are more likely to experience positive emotions and engage in the task with greater motivation. In the EFL context, academic self-efficacy serves as a pivotal belief system that shapes how learners evaluate the challenges they face and the strategies they employ to meet these challenges (Wang and Sun, 2024). Students with high academic self-efficacy are more confident in their language abilities and thus appraise their capabilities as well-suited to the demands of the classroom, leading to a heightened expectancy of action control (Byrd and Abrams, 2022). This, in turn, can lead to the arousal of positive achievement emotions, such as enjoyment and pride (Wang et al., 2021), which are conducive to a state of flow during language learning tasks (Dewaele and MacIntyre, 2022; Liu and Song, 2021). By fostering a strong sense of academic self-efficacy, educators can potentially enhance the value that learners place on language learning outcomes and bolster their perceived control over the learning process, thereby creating an environment where flow experiences are more likely to occur.

### 2.2 Flow experience in the EFL learning context

In the field of second language acquisition (SLA), the concept of flow stands out as a complex and dynamic psychological state that extends beyond the conventional boundaries of emotion or engagement. Flow is not merely an emotion, despite its intrinsic motivation and the pleasure derived from it. Nor is it simple engagement; rather, it is a complex, dynamic state of consciousness marked by total absorption, a merging of action and awareness, and a balance between perceived challenge and personal skill level (Csikszentmihalyi and Csikszentmihalyi, 2014). In the EFL context, flow is particularly significant as it represents a transformative learning experience. Learners who enter this state may find themselves at the nexus of challenge and skill, where the demands of the language task align with their competencies, leading to a loss of self-consciousness and a distortion of time perception (Mesurado et al., 2015). Building on the work of previous researchers, flow is recognized as an active and fluctuating mental state that is inherently enjoyable and intrinsically motivating. It is a state that can be productively absorbing and even potentially addictive (Mesurado et al., 2015), which speaks to its powerful role in driving learners towards engagement and achievement.

The cultivation of flow experience among EFL learners is a multifaceted endeavor that hinges on environmental and individual factors. Classroom environmental variables like task features are broadly discussed (Egbert, 2003). Researchers (Csikszentmihalyi et al., 2014; Egbert, 2003; Liu and Song, 2021) emphasizes the importance of clear goal setting, immediate feedback, and skill-challenge balance in tasks as facilitators of flow. Other scholars believe learners are more likely to encounter flow in groupwork tasks that foster their agency and provide a certain level of autonomy in task execution (Tao et al., 2022). Individual factors, such as the age of EFL learners, self-assessed proficiency level, attitudes towards English, and attitudes towards the teacher, can also play a significant role in fostering a state of flow (Dewaele et al., 2022). Furthermore, the emotional intelligence and personality traits of learners serves as a significant predictor of flow enhancement (Abdolrezapour and Ghanbari, 2022; Ljubin-Golub et al., 2018). These individual factors, which differentiate EFL learners’ flow experiences, may also be reflected in their demographic variables, such as age, academic years and majors. In the context of EFL learning, experiencing flow would facilitate students’ improved performance, augmented creativity, and positive affective states (Dewaele and MacIntyre, 2022; Li et al., 2021; Liu and Song, 2021). As such, it is imperative to understanding flow and exploring factors affecting flow in EFL contexts so as to enhance the learning experience and outcomes for EFL learners.

In the EFL learning contexts, researchers distinguish between two types of flow experiences: the general classroom flow and the task-specific flow. The former state is the experience of flow within the context of an EFL classroom, whereas the latter is an optimal emotional state induced by a task (Czimmermann and Piniel, 2016). Researchers conducted a thorough analysis of task-specific flow by examining the correlation between distinct language learning tasks and flow experience. For example, Aubrey (2017) explored how inter-cultural interaction in oral tasks enhance flow experience in Japanese EFL classrooms. Ghanbaran et al. (2023) investigated the role of task types and patterns in the perception of flow by Persian EFL learners when they were involved in communicative tasks. In Chinese EFL contexts, Li et al. (2021) investigated how the flow experience affected students’ digital game-based vocabulary learning. Chen et al. (2023) explored the effect of information presentation conditions on the flow experience of digital reading courses.

There are some fruitful studies exploring flow experience at a task-specific level, but few have set foot in the general EFL classroom context. Language learning in classroom settings usually includes various learning tasks and activities, and aims to cultivate EFL learners’ comprehensive language skills. Thus students may experience multiple task-specific flows in one class, and the latter task-specific flow experience may be influenced and reinforced by the former ones. Aubrey (2017) believed flow states are integrated by conditions influenced by multiple previous experiences but not the isolated one. Empirical results also proved that task-specific flow is closely related to classroom flow, and they have similar features and components as well (Czimmermann and Piniel, 2016). Hence it is critical to develop a holistic view of EFL learners’ classroom flow experiences, identify their levels of classroom flow, and propose:

H1: There are significant differences in the flow experiences of EFL learners based on gender, grade, and major.

### 2.3 Teacher support and its links with EFL learners’ flow experience

Teacher support is crucial in EFL learning since foreign language learning is a long, demanding, and challenging process (Zhang, 2019). Teacher support refers to a collection of resources provided by teachers consisting of emotional, cognitive, behavioral, and instrumental aspects to promote student accomplishment (Chen, 2005). Teacher support is a multi-faceted construct in the conceptual sense. Metheny et al. (2008) revealed three types of teacher support, they are instrumental, emotional, and informational support. Other scholars like Mercer et al. (2011) integrated the dimension of appraisal support into the construct of teacher support. Sadoughi and Hejazi (2022) conducted an empirical study among Iranian EFL learners and validated the four-dimensional construct of teacher support within the context of EFL learning. Emotional teacher support, encompasses the concern, affection, acknowledgment, and trust teachers extend to their students. Emotional support from teachers is paramount, as it addresses student’ affective needs and contributes to a positive learning environment. Researchers highlighted the importance of emotional engagement in language learning tasks, suggesting that it is a favorable condition for flow (Furrer et al., 2014; Amini and Amini, 2017). Instrumental support is related to providing materials, presenting challenging yet achievable tasks, and instructional guidance for EFL learners from a general perspective. By aligning task challenges with students’ skills and capabilities and offering clear learning objectives, teachers can significantly influence students’ flow experience (Nakamura and Csikszentmihalyi, 2009). In addition, this support helps students feel a sense of control over their learning process, which is a key component of flow (Csikszentmihalyi et al., 2014). Informational support entails the provision of information, guidance, or advice by teachers on specific language content and skills, which serves to improve students’ comprehension and mastery of language tasks, fostering autonomous learning particularly among top performers. Tennant et al. (2015) validated the linkage between teacher informational support and students’ academic engagement, while engagement is essential for achieving a state of flow (Ljubin-Golub et al., 2018). Appraisal support involves providing constructive feedback and recognition of students’ efforts, thereby addressing the psychological need for competency, relatedness and autonomy. When these psychological needs are met, students are more likely to become deeply engaged in the EFL learning process, leading to a state of classroom flow (Dincer et al., 2019).

Empirical evidence has demonstrated that teacher support is crucial in promoting EFL learners’ engagement (Sadoughi and Hejazi, 2021), while engagement, being a potential factor of flow, is responsible for inducing a state of flow (Shernoff et al., 2003). Recent empirical research has demonstrated that various forms of teacher support can effectively promote positive affective outcomes, such as enjoyment and pride, among EFL learners (Hejazi and Sadoughi, 2023; Sadoughi and Hejazi, 2021). Bakker et al. (2011) demonstrated that teachers’ autonomy-supportive communication styles, along with the corresponding performance feedback, exert a favorable influence on flow experience. Ljubin-Golub et al. (2020) also found that students who perceived a higher level of autonomy support from their teachers exhibited more frequent flow experiences during the learning process. The existing literature has not provided sufficient empirical data to establish a clear relationship between teacher support and flow experience in the context of university EFL learning. Furthermore, these studies have failed to distinguish between various forms of teacher support. Therefore, the present study aims to investigate four distinct types of teacher support and their relationship with flow experience within the Chinese EFL learning environment and propose:

H2: Teacher support has a significant positive impact on EFL learners’ flow experiences.

### 2.4 Academic self-efficacy and its role in teacher support and flow experience

Self-efficacy (SE), as defined by Bandura (1982), refers to an individual’s judgment of their capabilities to organize and execute the actions required to achieve expected performances. In academic settings, this concept is known as academic self-efficacy, which pertains to learner judgments about their ability to successfully achieve educational goals (Elias and MacDonald, 2007). Within the context of language learning, EFL learners’ academic self-efficacy is recognized as a significant influence on their performance across various language domains, including writing, reading and speaking (Shehzad et al., 2019). Learners with higher academic self-efficacy demonstrate greater confidence in tackling demanding tasks and are more likely to achieve a higher degree of language proficiency than those with lower self-efficacy (Daemi et al., 2017). Researchers have identified four key sources of academic self-efficacy: enactive learning experience, vicarious experience, verbal persuasion, and emotional and physiological states (Phan and Ngu, 2016). Enactive learning involves gaining insight through the direct experience of one’s own actions. For instance, in an EFL learning context, engaging students in enactive pre-reading activities can enhance their reading comprehension (Eshghipour and Khalili, 2016), leading to preferred learning outcomes and positive academic emotions in student. An empirical study conducted among Chinese EFL learners has also confirmed that the enactive learning experience, along with physiological and emotional states, serves as a significant predictor of their linguistic self-efficacy (Wang and Sun, 2024).

In the context of EFL learning, a study among Chinese EFL undergraduates revealed that students with higher self-efficacy are more likely to experience positive academic emotions, including enjoyment and pride (Wang et al., 2021). A recent study has also confirmed the importance of nurturing EFL learners’ self-efficacy in facilitating their flow experience (Jia et al., 2024). This suggests that students with greater self-efficacy tend to be more optimistic and confident, which can lead to a more positive emotional state and academic engagement (Putwain et al., 2013). Moreover, the mediating role of academic self-efficacy between teacher support and academic success has been established, further underscoring its importance in the academic domain (Gutiérrez and Tomás, 2019; Huang and Wang, 2023). Nevertheless, empirical investigations examining the linkage between teacher support and academic performance yield inconsistent results. Aldridge et al. (2012) found that the path coefficient of teacher support on academic efficacy in mathematics courses in UAE colleges was statistically insignificant. Daemi et al. (2017) revealed that there was a small correlation between EFL learners’ perception of teacher support and their reflected academic self-efficacy. While some Chinese scholars (e.g., Chen et al., 2020; Huang and Wang, 2023; Liu et al., 2018) consented that teacher support can significantly enhance university students’ academic self-efficacy. Focusing on the EFL learning field, Zheng et al. (2017) revealed teacher support has a strong impact on learners’ confidence in their English language performance, which indicates teacher support may have links to the boost of academic self-efficacy. Thus, we propose:

H3: Academic self-efficacy plays a mediating role in the relationship between teacher support and classroom flow within an EFL learning context.

## 3 Methodology

### 3.1 Participants and procedure

This study employs a cluster sampling method to survey among students who enrolled in the course “College English” at an application-oriented university in central China. “College English” is a compulsory course for non-English majors in most universities in China, which aims to improve EFL learners’ comprehensive English proficiency and English skills in listening, speaking, reading, writing and translation. Generally, the course was offered to freshmen and sophomores, hence, the research focused its participants on the undergraduate students of grade one and grade two.

To minimize the impact of extraneous variables, the study targeted six College English teachers with comparable professional backgrounds and teaching styles. All six teachers hold lecturer titles or above, are around 40 years old, and have teaching experience of over 10 years. They each teach mixed classes with approximately 100 students. The survey was strategically administered by the lead researcher during the 10-min breaks of each teacher’s class, with the consent of both teachers and students. This timing was selected because it ensured that students’ perceptions of teacher support and classroom flow were still vivid and genuine, immediately following their English class. Additionally, the brief survey interval prevented disruption to subsequent class activities. Students are given an online survey link generated by the Wenjuanxing platform, a Chinese version of SurveyMonkey. Before their filling out the composite questionnaire, the researcher stated the introduction of the research and matters needing attention. Specifically, they are assured that the data collected would be used only for research purposes, and their scores have nothing to do with their English academic performance evaluation. The survey adheres strictly to the principles of voluntariness and anonymity. The participants signed the informed consent of the online questionnaire. A total of 602 questionnaires were collected, and the questionnaires with many identical answers and submitted in less than 1 min were removed. The final number of valid responses is 557 (including 361 males and 196 females; 251 first-year and 306 s-year students; 472 students from science and engineering majors, and 85 from humanities and social sciences majors) and were analyzed to address the RQs using SPSS and Amos software. The data were collected from October 15th to October 29th in the year of 2023.

### 3.2 Instruments

Despite English being a second language for the participants, their proficiency levels and comfort with the language varied widely. To accommodate this diversity, a bilingual English-Chinese version of the survey was provided, allowing participants to respond in the language they felt most at ease with. The translation process involved an initial conversion of all survey items into Chinese by a reputable translation software. Subsequently, an experienced English instructor from the same college where the data were collected meticulously reviewed and refined the translations to ensure linguistic accuracy and cultural sensitivity. All the items were arranged on a 5-point Likert scale from “1” (strongly disagree) to “5” (strongly agree).

#### 3.2.1 Classroom flow

The Flow Short Scale (FKS), developed by Vollmeyer and Rheinberg (2006), and inspired by six characteristics of flow, including action-awareness merging and a sense of control (Csikszentmihalyi, 1988), was adapted for this study to measure the classroom flow experienced by EFL learners. The adaptation involved minor modifications to ensure the scale’s suitability for the Chinese university EFL learning context. The scale consists of 10 items, two of which are exemplified here: “I feel just the right amount of challenge in my English class” and “I feel that I have everything under control in my English class.” Previous validation work with this scale among Chinese undergraduates demonstrated a high level of internal consistency, with a Cronbach’s alpha of 0.825 reported by Gao and Zhang (2023). In the current study, the Cronbach’s alpha for the FKS was even higher at 0.949, indicating a very strong reliability of the scale in assessing the classroom flow of EFL learners.

#### 3.2.2 Teacher support

Foreign Language Teacher Support Scale (FLTSS) (Sadoughi and Hejazi, 2022) was adapted to measure teacher support perceived by EFL learners. It consists of four sub-scales to measure different dimensions of perceived teacher support, namely, emotional, instrumental, appraisal, and informational. The emotional dimension comprises 5 items, the sample item is “My English teacher really understands my feelings.” The instrumental dimension comprises 7 items, the sample item is “My English teacher gives me opportunities to express opinions.” The appraisal dimension comprises 6 items, the sample item is “My English teacher provides me with valuable feedback on my performance.” The informational teacher support comprises 7 items, the sample item is “My English teacher provides me with more examples/explanations when I do not understand a point.” In the current research, the value of Cronbach’s alpha is 0.908, 0.946, 0.965 and 0.964, respectively.

#### 3.2.3 Academic self-efficacy

In this study, academic self-efficacy among university EFL learners was assessed using the adapted and EFL-contextualized version of the Motivated Strategies for Learning Questionnaire (MSLQ) as developed by Zheng et al. (2017). The shortened form of the questionnaire comprises 5 items, and has been validated among Chinese university EFL learners (Cronbach’s alpha = 0.83) in Zheng et al.’s (2017) research, which implies the applicability of the scale in the current study. In the present sample, the scale also shows high reliability (Cronbach’s alpha = 0.954).

## 4 Results

### 4.1 Common method bias test and normality test

It is imperative to test common method bias for the self-report data. Multi-factor model CFA and single-factor model CFA were used to compare the model fitting. The results showed that the multi-factor model χ^2^ = 3204.149, *df* = 725, △χ^2^ = 5100.093, △*df* = 15, *p* < 0.001 was significantly lower than the single-factor model, and the two models were significantly different (Richardson et al., 2009). Therefore, it is considered that the data in this study are not affected by the common method bias, and the relationship between the variables derived from the data is credible. Normality tests were also conducted. The absolute value of the Skewness test was between 0.022 and 1.325, the absolute value of the Kurtosis test was between 0.047 and 2.257, Mardia value was 908.591, which was smaller than P*(P + 2) = 1,680 (P was the number of observed variables), which indicate that the total scores of all the variables in samples were normally distributed, and subsequent parametric tests can be carried out.

### 4.2 Descriptive analysis and difference analysis

To address the first RQ, a series of descriptive analyses was conducted. The average score of EFL learners’ classroom flow is 3.38, which closely aligns with the results of another empirical research conducted in China whose mean score of Chinese undergraduates’ language flow in blended learning is 3.27 (Wang and Huang, 2022), but falls short of the results observed among English major students at a Hungarian university whose mean score of classroom flow is 3.53 (Czimmermann and Piniel, 2016). Notably, 67.7% of participants scored higher than 3 points.

Independent sample t-test was used to examine whether there are significant differences in demographic variables (gender, grade and major). The results indicate that there is a significant differences in classroom flow between boys and girls (*p* < 0.01), and the mean score of boys’ classroom flow (*M* = 3.460, *SD* = 0.944) is significantly higher than that of girls. In addition, the results indicate that the mean score of classroom flow of second-year students (*M* = 3.603, *SD* = 0.920) is significantly higher than that of freshmen. In line with this finding, second-year students exhibit a significantly higher average score in teacher support (*M* = 4.104) compared to freshmen (*M* = 3.658), suggesting a positive correlation between the level of teacher support and the students’ flow experience in the EFL learning process. However, no significant differences were observed in classroom flow among students of various majors (*p* > 0.05). Thus H1 received partial support. The results are displayed in Supplementary Table S1.

### 4.3 Construction structural equation model

According to the recommendations of Anderson and Gerbing (1988), structural equation modeling analysis should be conducted in two stages: the first stage involves performing confirmatory factor analysis on each research dimension and measurement item to assess the reliability, convergent validity, and discriminant validity of each dimension. The second stage involves reducing multiple measurement items to a few key indicators and then conducting path analysis on the structural model to test the hypotheses in the study.

#### 4.3.1 CFA analysis

To address the second and the third RQ, structural equation modeling was conducted to test the causality between the variables and maximum likelihood estimation was used to estimate the relevant parameters. First, confirmatory factor analysis was used to test the questionnaire to ensure the validity and reliability of the research. The results showed that the Squared Multiple Correlation (SMC) ranged from 0.479 to 0.925; Factor load was between 0.537 and 0.962; The correlation coefficient was between 0.747 and 0.872; The CR values for teacher support, academic self-efficacy, and flow experience are 0.971, 0.955, and 0.951, respectively, and the AVE values are 0.892, 0.809, and 0.661, indicating that the AVE for each construct is greater than the square of the inter-correlation coefficients of the constructs, which suggests good convergent validity for the scales (Fornell and Larcker, 1981; Slater et al., 2007); Absolute fit index: Chi-square freedom ratio was 4.508, RMSEA = 0.079, AGFI = 0.727; Additional adaptation indicators: NFI = 0.881, TLI = 0.89; Thin provisioning indicators: PNFI = 0.828, PCFI = 0.851, which belongs to the moderate correlation. All indexes were within the acceptable range, indicating that the model had a good fit (Hair et al., 2019) as shown in Supplementary Figure S1.

#### 4.3.2 SEM path analysis

After testing the reliability and validity, a second-order structural equation model was constructed using teacher support, academic self-efficacy, and flow experience. The fit indices showed: SRMR = 0.046, RMR = 0.049, PGFI = 0.675, RFI = 0.874, IFI = 0.905, CFI = 0.905. The overall model fit is good.

After the fit test, path hypothesis testing was conducted. Amos 26.0 was used to establish the direct path model of teacher support → flow experience and the intermediary model of teacher support → academic self-efficacy → flow experience, respectively, and the parameter values in the model were used to respond to the research questions. The bootstrapping method was used to test the effect of each path. If the 95% confidence interval of the intermediate effect obtained by repeated sampling does not contain 0, it means that the path effect reaches a significant level of *p* < 0.0.5 (Shrout and Bolger, 2002). The results show that the path coefficient of teacher support on classroom flow experience is 0.747 (*p* < 0.001, 95% CI [0.684, 0.803], not including 0), indicating that teacher support has a significant positive predictive effect on EFL students’ flow experience. H2 was supported.

In addition, to distinguish the impact of each of the four dimensions of teacher support in both the direct path and the mediation model, individual mediation models were established for each dimension, respectively. The findings revealed that the largest mediating effect was observed in instrumental teacher support (*β* = 0.737, *p* < 0.001), followed by emotional teacher support (*β* = 0.725, *p* < 0.001), informational teacher support (*β* = 0.684, *p* < 0.001) and appraisal teacher support (*β* = 0.634, *p*<0.001). The path analysis results also indicated that different types of teacher support have different predictive effects on EFL learners’ classroom flow. Among them, appraisal teacher support has the strongest predictive power on classroom flow (*β* = 0.751, *p* < 0.05), while emotional teacher support has the weakest predictive effect on classroom flow (*β* = 0.669, *p* < 0.01), as shown in Supplementary Table S2.

Additionally, the direct effect of teacher support on flow experience is 0.198 (*p* < 0.001, 95% CI [0.107, 0.294], not including 0). The indirect effect was calculated as 0.549 (0.762*0.721) (*p* < 0.001, 95% CI [0.465, 0.634], not including 0), and the model’s explained variance increased by 19.7%, as shown in Supplementary Table S3. The results indicated that academic self-efficacy exhibited a partial mediating role between teacher support and classroom flow experience. H3 was supported, as shown in Supplementary Figure S2.

## 5 Discussion

### 5.1 An overview of students’ classroom flow experience

Using Egbert’s (2003) criterion, participants scoring above 3.0 on the five-point classroom flow scale were considered to have experienced flow. In the present study 67.7% EFL learners in the sample university have experience classroom flow accordingly, which is smaller than the proportion of students experiencing flow in Hungarian university’s EFL classrooms, whose percentage is 74% (Czimmermann and Piniel, 2016). Although the mean score and the percentage of students’ classroom flow are lower than those of their Hungarian peers, the results indicate a high flow in the observed EFL classrooms. Possible reasons for this discrepancy include the following. First, the EFL learning class size in Chinese universities is larger, thus may lead to the distraction of some students’ learning attention. Second, students surveyed in Hungarian universities were English major students, and thus they have a better English language foundation and more motivation to learn English compared to the Chinese EFL learners in the present study. Third, the teaching methods and resources used in the two contexts might differ, which could affect the students’ classroom flow experience.

The results of the t-test show that there is a significant gender difference in university EFL learners’ classroom flow experience, and the level of male students’ classroom flow is significantly higher than that of female students. Furthermore, the degree of teacher support perceived by boys was significantly higher than that of girls, which is in line with some previous research (e.g., Zhang et al., 2020). One possible explanation for this discrepancy is that the teaching strategies employed by English instructors may cater to the perceived needs of the majority, potentially overlooking the superior English proficiency and foundational skills of female students. As a result, female students, who are often more intrinsically motivated and dedicated to language learning (Mohammadi and Sharififar, 2016; Oga-Baldwin, 2019), might perceive the instructional challenges as mismatched with their abilities, which could impede the challenge-skill balance crucial for achieving a flow state (Aubrey, 2017). Moreover, the sense of control is a pivotal element in the flow experience (Czimmermann and Piniel, 2016). It is plausible that female students may experience heightened anxiety and a sense of being overwhelmed if they feel a loss of control during EFL classroom activities, which could, in turn, negatively affect their flow experience.

Furthermore, the present investigation uncovered substantial disparities in classroom flow among freshman and sophomore students, indicating a strong link between classroom flow and English proficiency level. This implies that as students’ English proficiency improves, their adjusted challenge-skills balance also increases, thereby enhancing their likelihood of experiencing flow in EFL classrooms. This conclusion aligns with prior research emphasizing the crucial role of language proficiency in promoting flow experiences (e.g., Cho, 2018; Dewaele and MacIntyre, 2022).

### 5.2 The relationship between teacher support and classroom flow

The current study found teacher support has a significant positive predictive effect on EFL learners’ classroom flow experience, supporting previous findings that highlighted the correlation between teacher support and positive academic emotions (e.g., Liu et al., 2018; Sadoughi and Hejazi, 2021). This indicates that teachers play a crucial role in promoting students’ engagement and emotions in classroom activities. A deeper examination of the relationship between teacher support dimensions and classroom flow shows that appraisal teacher support exerts the most substantial predictive influence. Appraisal teacher support helps learners track their learning process and regulate their learning strategies by interpreting teachers’ feedback and formative assessment (Nicol and Macfarlane-Dick, 2006), and also satisfies students’ needs for autonomy in learning behavior (Deci and Ryan, 1987). The results thereby validate the applicability of self-determination theory in the context of EFL learning. Informational teacher support also has robust predictive power on EFL learners’ classroom flow. This type of support is considered a good scaffolding in language learning by offering students extra resources, good examples and clear explanations, and providing them with useful guidelines for improving their English (Sadoughi and Hejazi, 2022). When teachers use more autonomy-supportive instructional behaviors like offering scaffolding to students and using frequent inquiry settings, students devote more engagement and concentration to their learning, thus facilitating a state of flow (Reeve et al., 2004; Borovay et al., 2019). While instrumental and emotional teacher support also predict classroom flow, their effects are less pronounced than those of appraisal and informational support. This may be because EFL learners at university levels might already possess a high degree of autonomy and motivation, thus they seem to require less hands-on guidance and emotional encouragement to achieve a state of flow. Moreover, the nature of university-level EFL courses, which require learners to engage actively with the language, thinking critically and creatively about the content, leading learners to more autonomous learning and thus enter into a state of flow more naturally.

### 5.3 The mediating effect of academic self-efficacy

The present study revealed that academic self-efficacy has a mediating effect between teacher support and classroom flow. This finding is in line with previous research indicating that teacher support can enhance students’ academic self-efficacy (Liu et al., 2018; Mercer et al., 2011). It suggests that teachers’ encouragement and guidance play a vital role in fostering students’ academic self-efficacy beliefs, which ultimately contribute to their engagement and flow in the classroom. Additionally, the findings indicated a positive correlation between academic self-efficacy and the experience of flow within the classroom, which aligns with prior research that highlighted the predictive role of self-efficacy in the occurrence of flow experience during EFL learning contexts (Jia et al., 2024). This suggests that students with higher self-efficacy beliefs are more likely to show greater engagement and persistence that is necessary to achieve academic goals, and thus are more likely to experience flow in their learning process. The results also validate the control-value theory of achievement emotions in EFL contexts. Students possessing a high level of academic self-efficacy accurately assess their individual abilities in relation to the given situation and challenges. This assessment leads to the formation of action-control expectancies, thereby triggering activity-related emotions that are intricately intertwined with the flow experience (Pekrun, 2006). Of note, instrumental and emotional teacher support play crucial role in fostering students’ academic self-efficacy, which means teachers’ provision of effective learning resources and strategies, their emotional care and encouragement can help to stimulate students’ academic self-efficacy beliefs, and ultimately inspire EFL learners’ positive emotions and engagement in the learning process.

## 6 Conclusion, implications and limitations

In conclusion, this study investigated the relationships among teacher support, academic self-efficacy, and classroom flow in Chinese university EFL learners. The findings suggest that the majority of EFL learners in Chinese universities experienced classroom flow, and the level of classroom flow exhibited significant differences across gender and grade. The findings also suggest the independent and joint effects of teacher support and academic self-efficacy in fostering EFL learners’ flow experience, and different predictive effects of distinctive teacher support on facilitating EFL learners’ classroom flow. In addition, this study has theoretically confirmed the applicability of social support theory and control-value theory in the field of second language acquisition, and provided empirical support for these two theories. The results highlight the importance of teachers’ role in promoting students’ engagement and flow experiences, as well as the significance of fostering students’ academic self-efficacy beliefs.

The findings highlight the importance of integrating external and internal factors with positive emotions in EFL learning, which provide useful implications for EFL practitioners, educators, learners as well as researchers. Firstly, it is crucial to address the gender difference in classroom flow experience by implementing gender-specific interventions for EFL learners. Secondly, teachers should aim to create engaging and challenging learning tasks, providing more targeted teaching contents that cater to the diverse proficiency levels of their students, thus facilitating more flow experience. That is to say, teachers should fully consider students’ individual differences during the teaching process and customize their teaching methods accordingly. It is also expected to provide a supportive and inclusive environment in EFL classrooms to enhance students’ flow experience. Teachers should exhibit different types of support in different situations to meet students’ learning psychological needs for autonomy, competence and relatedness (Tao et al., 2022). Appraisal teacher support and informational teacher support should be reinforced in university EFL learning contexts. Constructive feedback that acknowledges students’ efforts and provides clear guidance on how to improve can significantly boost their confidence and motivation. Moreover, timely and specific feedback and guidance can help students identify areas where they need to exert more effort, thus fostering a sense of control over their learning process, and improving self-efficacy beliefs. In addition, the findings provide practical implications for facilitating the experience of EFL learners’ flow by cultivating their self-efficacy. It is essential for teachers to nurture a growth of academic self-efficacy among their students. This can be achieved by promoting collaborative learning strategies, because cooperative and trusting relationships among peers can boost students’ confidence in learning (Xu and Tu, 2023). Teachers are also advised to emphasize the idea that skills and abilities can be developed through dedication and hard work, students hence may be more willing to embrace challenges and persevere through difficulties, thus cultivating higher academic self-efficacy and positive academic emotions.

Though the current research is an addition to the field of positive emotions in Language acquisition, we need to acknowledge some limitations here to promote future research. Firstly, although our findings were obtained in a large-scale sample of EFL learners, it does not necessarily mean that the findings can be generalized to the whole EFL population in China considering various complex contextual factors. Secondly, the study conducted a cross-sectional investigation of the focal issue without considering the fact students’ perceptions about teacher support and classroom flow might change over time. Thirdly, the study did not control for other possible factors that might influence classroom flow, such as student motivation, classroom environment, and teacher effectiveness. Future research could address these limitations by conducting experimental or longitudinal studies with diverse samples and controlling for additional factors. Moreover, future studies could be conducted to investigate the duration and stability of flow experiences in EFL learning contexts over time, as well as the other potential predictive factors of flow experiences. By doing so, we can gain a more comprehensive understanding of the complex nature of classroom flow and its implications for EFL learning outcomes.

## Data Availability

The raw data supporting the conclusions of this article will be made available by the authors, without undue reservation.
