# Supplementary material for: Teacher support as predictors of Chinese EFL learners’ classroom flow: the mediating role of academic self-efficacy
Source: Front Psychol. 2024 Sep 27;15:1452146. doi: 10.3389/fpsyg.2024.1452146 (PMC11466759; doi:10.3389/fpsyg.2024.1452146)
Supplement: Supplementary file 1 [file Table_1.DOCX]

Supplementary Material

# Supplementary Figures and Tables

**Table 1 Descriptive statistics and results of independent sample t-test**

|  | | Classroom flow(CF) | | |
| --- | --- | --- | --- | --- |
|  |  | *M* | *SD* | *t* |
| Gender | male | 3.460 | 0.944 | 2.760** |
|  | female | 3.236 | 0.857 |  |
| Grade | fresh | 3.111 | 0.845 | -6.574*** |
|  | sophos | 3.603 | 0.920 |  |
| Major | sci-engi | 3.407 | 0.921 | 1.567 |
|  | hum-soci | 3.238 | 0.901 |  |

Note: 1. fresh: freshman; sophos: sophomore; sci-engi: science and engineering; hum-soci: humanities and social sciences;

1. number _males_= 361, number _females_= 196;number _fresh_= 251, number _sophos8_= 306;number _sci-engi_= 472, number _hum-soci_= 85;
2. **p*<0.05, ***p*<0.01, ****p*<0.001.

**Table 2 Path analysis of teacher support on classroom flow**

|  | Estimate | *p*-value | Confidence Interval |
| --- | --- | --- | --- |
| TS → CF | 0.747 | .000 | [0.684, 0.803] |
| ETS → CF | 0.669 | .009 | [0.587, 0.735] |
| IntS → CF | 0.670 | .002 | [0.575, 0.738] |
| ATS → CF | 0.751 | .010 | [0.692, 0.803] |
| InfS → CF | 0.711 | .009 | [0.646, 0.767] |

Note: TS: teacher support; CF: classroom flow; ETS: emotional teacher support; InsTS: instrumental teacher support; ATS: appraisal teacher support; InfS: informational teacher support

**Table 3 The mediating effect of academic self-efficacy**

|  | Estimate | *p*-value | Confidence Interval | R^2^ |
| --- | --- | --- | --- | --- |
| Direct effect |  |  |  |  |
| TS → CF | 0.198 | .000 | [0.107, 0.294] |  |
| TS → AS | 0.762 | .000 | [0.694, 0.818] |  |
| AS → CF | 0.721 | .000 | [0.609, 0.810] |  |
| Indirect effect |  |  |  |  |
| TS → AS → CF | 0.549 | .000 | [0.465, 0.634] | .777 |
| Total effect |  |  |  |  |
| TS → CF | 0.747 | .000 | [0.684, 0.803] | .580 |

Note: TS: teacher support; CF: classroom flow; AS: academic self-efficacy

**Figure 1 Confirmatory factor analysis diagram**

**
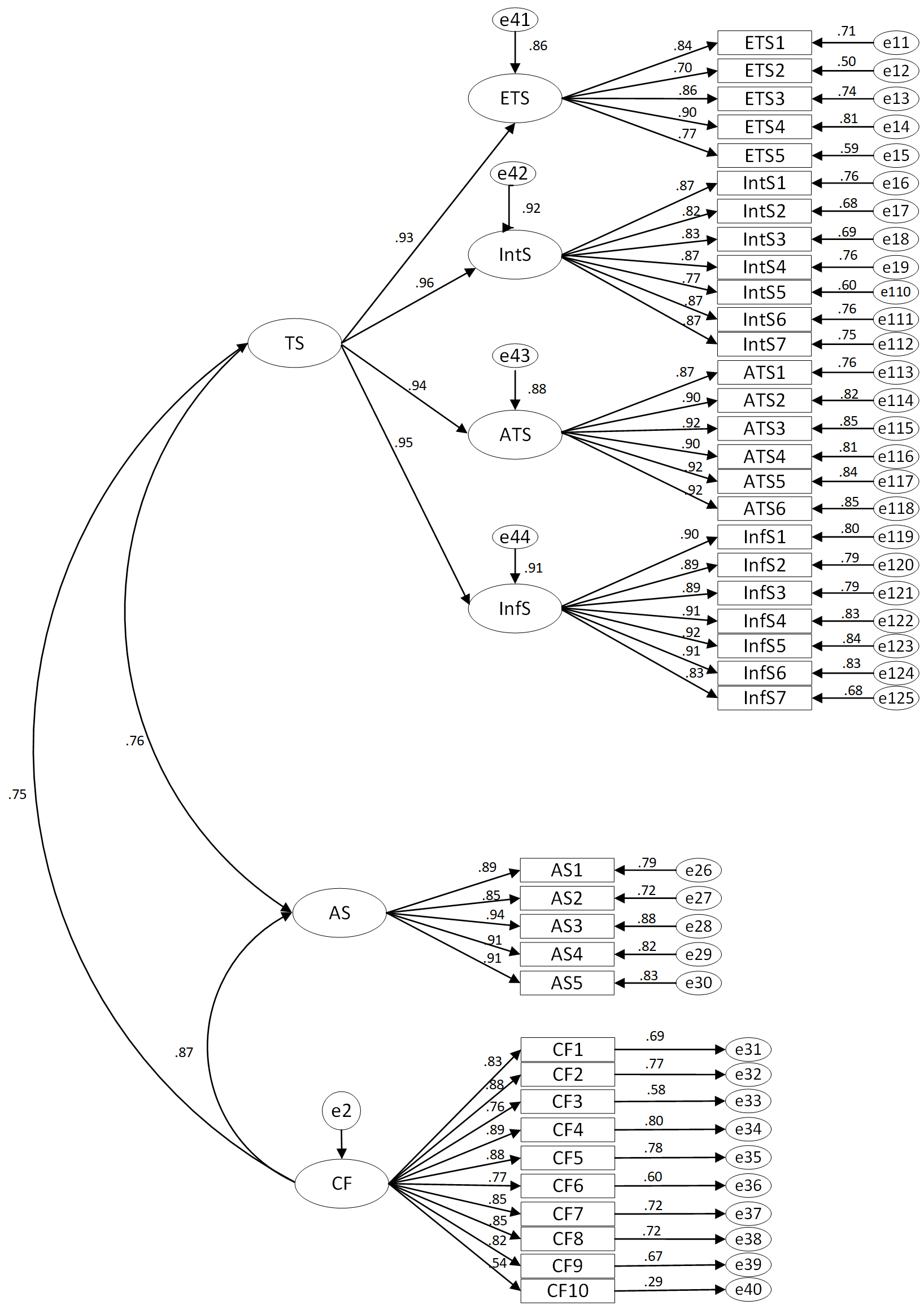
**

**Figure 2 Structural equation model with mediating variable**

**
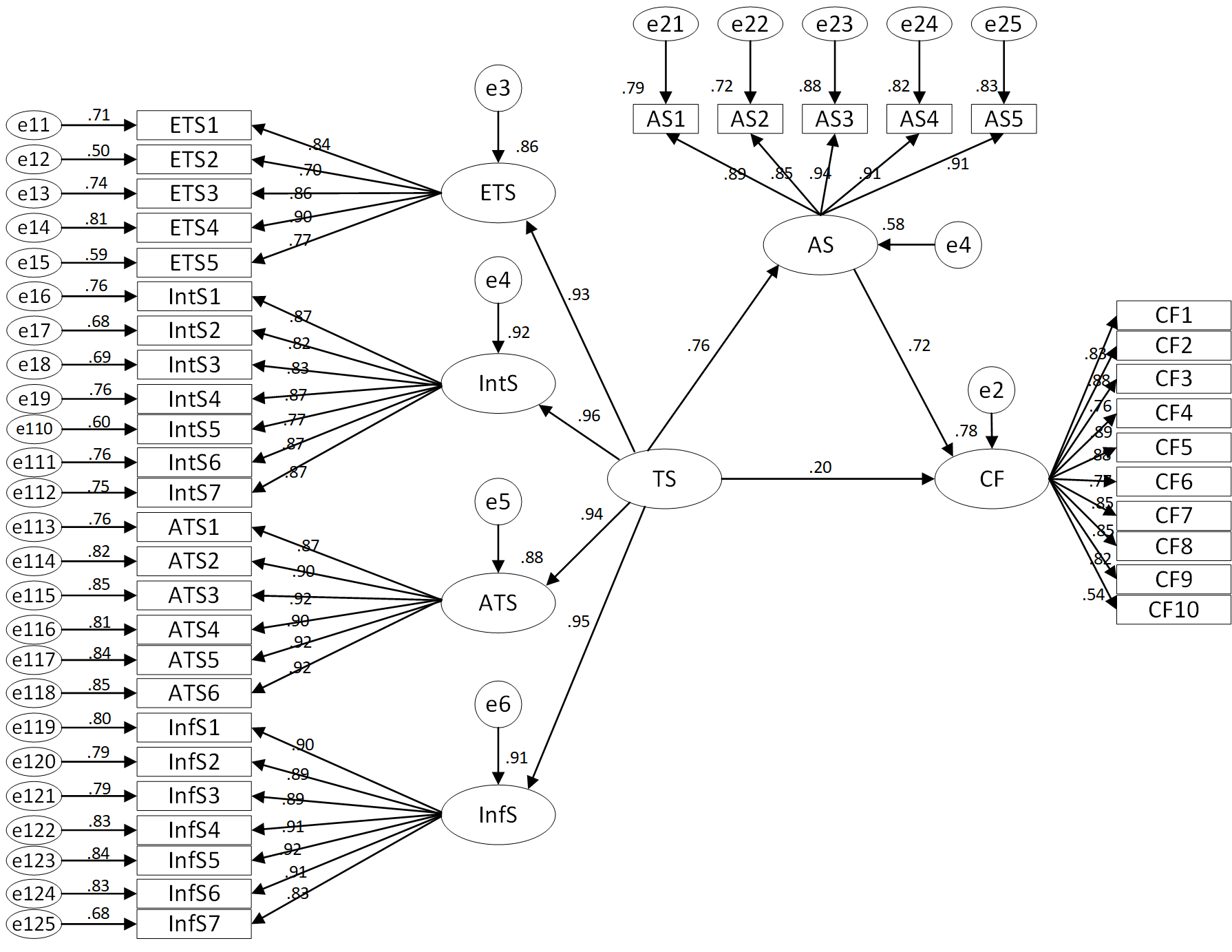
**
